# P-1549. Understanding Epstein-Barr Virus and other Infectious Drivers of Hemophagocytic Lymphohistiocytosis: A Single-Center Study

**DOI:** 10.1093/ofid/ofaf695.1729

**Published:** 2026-01-11

**Authors:** Amber Feng, Julian J Weiss, Andrea Sitlinger, Julia A Messina, Megan Hansen, Jie Wang

**Affiliations:** Duke Hospital, Durham, North Carolina; Duke University Hospital, Durham, North Carolina; Duke University, Durham, North Carolina; Duke University, Durham, North Carolina; Duke University Hospital, Durham, North Carolina; Duke University Hospital, Durham, North Carolina

## Abstract

**Background:**

Hemophagocytic lymphohistiocytosis (HLH) is a rare but morbid condition. HLH can be driven by malignancy, autoimmune disease, infection, and/or genetic predisposition. Epstein-Barr virus (EBV) is an oncogenic virus that is commonly associated with HLH, but the drivers and outcomes of both EBV-HLH and non-EBV associated HLH are incompletely understood.

**Figure:**
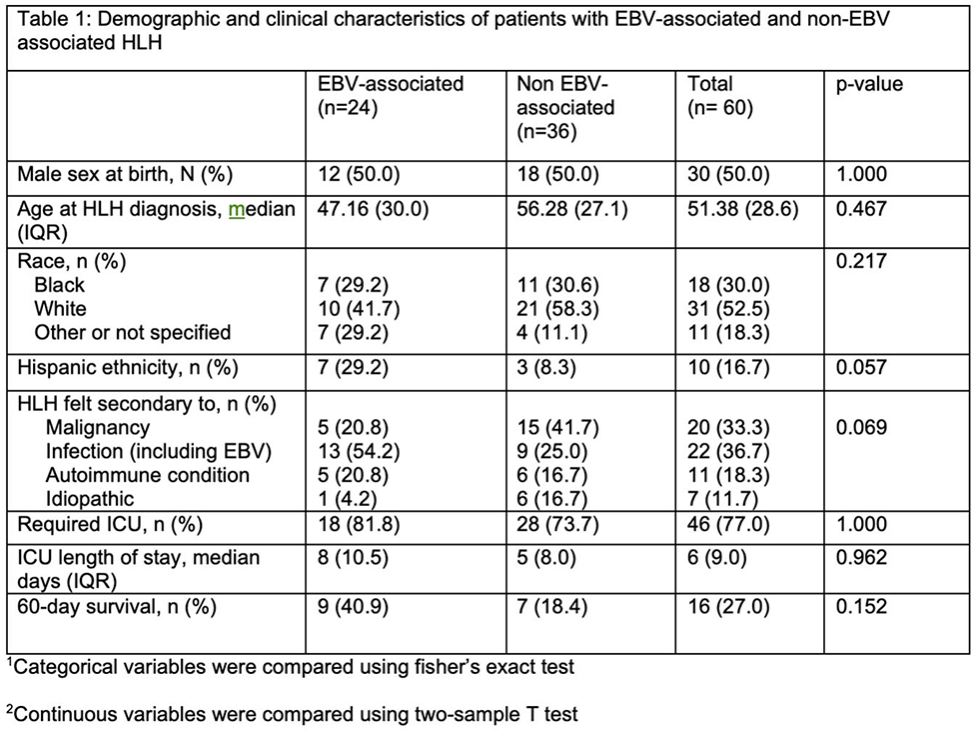

Epstein-Barr Viral Loads since admission

**Figure 1:**
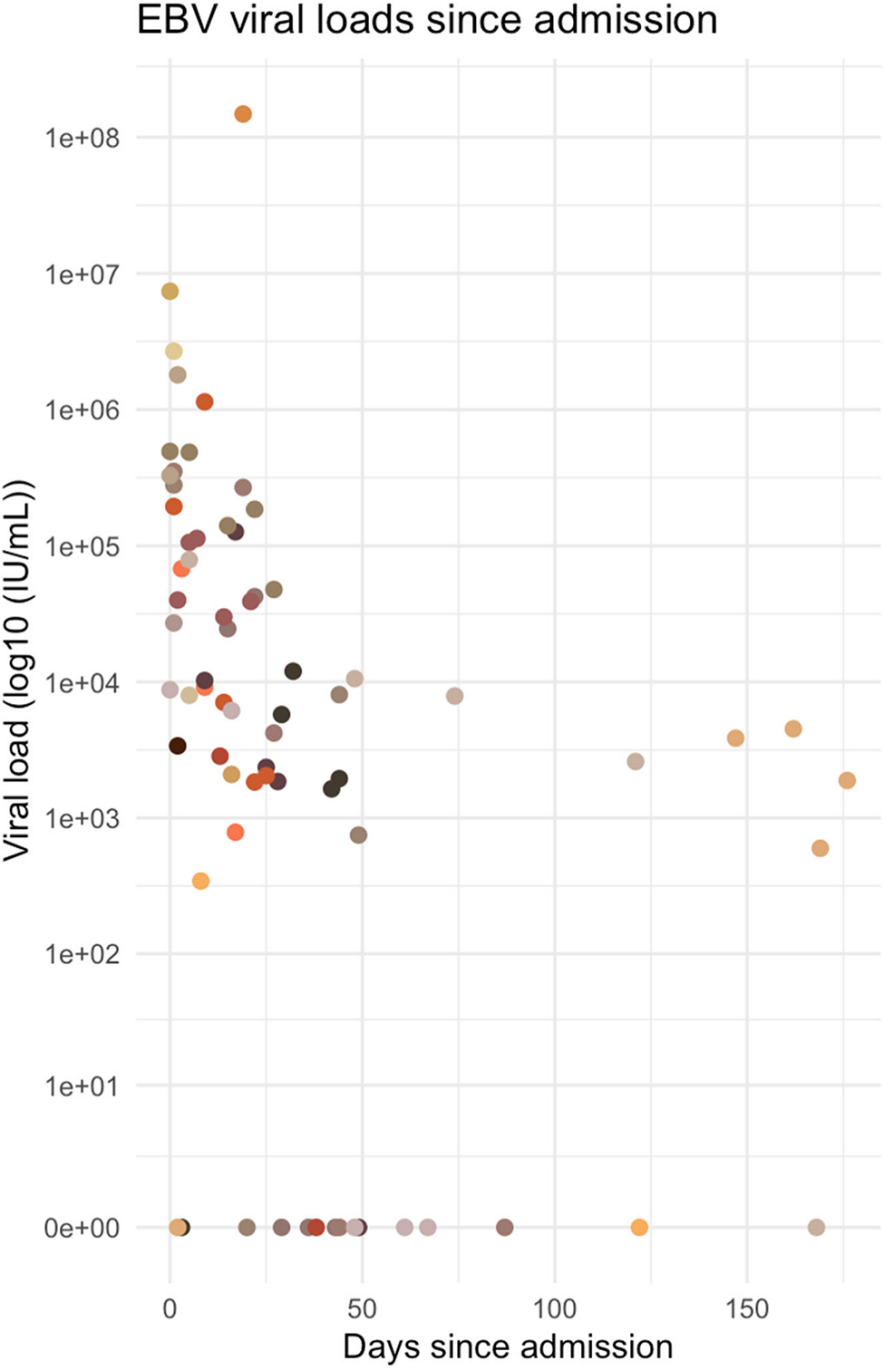
EBV viral loads for each individual patient who had an EBV viral load above the limit of detection during their admission mapped by days since admission. Each different color represents each individual patient. One patient in our dataset had a viral load measured in copies/mL while the rest were measured in IU/mL. For the purpose of this figure, the patient with a viral load in copies/mL was excluded.

**Methods:**

We conducted a retrospective review of adult patients at Duke University Hospital diagnosed with HLH between 01/01/2013 and 12/31/2024 through a SlicerDicer search of the electronic medical record. The diagnosis of HLH was verified using HLH-2004 diagnostic criteria. Patients were categorized as “EBV-HLH” if they had an EBV viral load above the limit of detection. Data on demographics, outcomes, and underlying HLH trigger were collected for the EBV-HLH and non-EBV associated HLH cohorts. Two-sample t-tests were performed to assess significance of continuous variables and Fisher’s exact tests for differences between categorical variables, with a p-value >0.05 being statistically significant.

**Results:**

We identified 60 patients with a diagnosis of HLH during the 10-year study period. Of these patients, 24 (36.7%) had EBV-HLH. There were no statistically significant differences in age, sex, or race between the two groups, though the EBV-HLH cohort had a higher proportion of Hispanic patients. Fifty-four percent of patients in the EBV-HLH cohort and twenty-five percent of patients in the non-EBV-HLH cohort had an infection as the primary driver for their HLH. Infections were variable and included viral etiologies (EBV, cytomegalovirus, COVID-19), gram-negative bacteremia, and less common infections such as Ehrlichiosis. There were no significant differences in outcomes including admission to the intensive care unit (ICU), ICU length of stay, or 60-day mortality between groups.

**Conclusion:**

At our institution, we found that EBV-HLH represents a significant portion of all patients with HLH. Underlying infectious drivers play a significant and likely underrecognized role in both EBV-HLH and non-EBV associated HLH. There was a higher proportion of Hispanic patients who had EBV-HLH compared to non-EBV HLH, raising need for further study on potential genetic or environmental risk factors.

**Disclosures:**

Andrea Sitlinger, MD, BeiGene: Advisor/Consultant|DAVA Oncology: Honoraria|Genmab: Grant/Research Support|Loxo Oncology: Grant/Research Support Julia A. Messina, MD, MHS MS, Seres: Advisor/Consultant|UpToDate: Royalties

